# Directions for sex and gender-based health research in Korea: implications of the Amendments of the Framework Act on Science and Technology

**DOI:** 10.4069/kjwhn.2022.12.14

**Published:** 2022-12-29

**Authors:** Heisook Lee

**Affiliations:** 1 GISTeR (Korea Center of Gendered Innovations for Science & Technology Research), Seoul, Korea; 2 Department of Math, Ewha Womans University, Seoul, Korea

## Introduction

Since the first report, “Gendered Innovations: How Gender Analysis Contributes to Research,” published by the European Commission in 2013, many examples and case studies have demonstrated that the integration of sex and gender-based analysis (SGBA) into research can not only enhance research excellence but also add inclusive value for both men and women [1]. In the coronavirus disease 2019 (COVID-19) pandemic, the incorporation of SGBA into research has received new attention as extensive scientific evidence has indicated that biological sex matters in the immune system, which responds differently to vaccination in males and females. The publication of scientific evidence that does not properly reflect SGBA not only leads to unequal research results in men and women but can also have life-threatening results and cause investment losses.

In order to reach a higher level of excellence in biomedical and health research and development, reflecting on SGBA has been an international trend, and various research support policies have been established and applied in the European Union (EU), the United States (US), and Canada [2]. The US National Institute of Health (NIH) mandated “integrating sex as a biological variable in vertebrate animals and human studies” in 2016 and the EU recommended reflecting gender as a dimension in research during Horizon 2020 for 7 years (2014–2020) and carried out “Gender Flagged” projects [3]. Horizon Europe, which began in 2021 following Horizon 2020, further introduced a research and innovation policy in which the integration of the gender dimension into research and teaching is recommended in addition to the mandatory process-related requirements for a gender equality plan [4].

An SGBA policy of peer-reviewed journals is another factor in promoting gender-based research in health. More than 33 professional journals in the field of biomedical science and health research such as *Nature, The Lancet*, and *Cell* have introduced policies requiring or strongly recommending SGBA analysis when submitting manuscripts [5].

## Current status of integrating sex and gender-based analysis into research

To find out how well SGBA has been integrated into research wherever applicable, a study [6] analyzed journals indexed in Web of Science from 2010 to 2021 in the field of biomedical and health science, using the following search terms: “sex factor*” OR “sex characteristic*” OR “sex difference*” OR “gender factor*” OR “gender characteristic*” OR “gender difference*” not “sex* partner*” OR “sex* selection*” OR “sex* behavior*” OR “sex* behaviour*” (data downloaded on August 2, 2022). Out of a total of 7,899,483 published papers, only 60,562 papers (0.77%) contained the keywords sex and gender. The field of neurosciences had the highest proportion of papers integrating SGBA, at 10,021 papers out of 476,180 papers (2.10%), followed by the fields of hormone-metabolic diseases at 3,810 papers out of 219,398 (1.74%) and cardiovascular diseases at 3,043 papers out of 247,731 (1.23%). However, although pharmaceutical studies triggered the integration of SGBA into research because of sex differences the adverse drug reactions, the proportion of papers integrating SGBA was only 0.57% (2,925 papers out of 514,882). Furthermore, in the field of immunology, in which SGBA should be actively integrated into research in the context of the COVID-19 pandemic [7,8], only 0.35% of research papers (1,027 papers out of 297,159) considered sex and gender differences. Given that research integrating SGBA in biomedicine and health is still very low, there is a risk that skewed data or knowledge from research may lead to further bias in medical artificial intelligence (AI). The health chatbot developed by Babylon Health is an example [9,10]. This chatbot participated in the United Kingdom’s medical license exam, scoring higher than the average physician, and was advertised to be able to make diagnoses 40% faster than humans, but was accused of gender discrimination. When presented with a hypothetical 59-year-old man and woman with the exact same habits, including smoking and drinking, who complained of chest pain and nausea, the chatbot advised the man to go to the emergency room immediately, whereas the woman was instructed to consult a family doctor if the symptoms do not improve after a few days. This is because the chatbot judged that men were at risk for heart attack and women were at risk for depression or panic disorder, which could have been due to the use of biased scientific data accumulated from long-standing practices and prejudices that heart disease is a mainly male problem [1].

In the era of digital transformations, serious consideration should be given to whether bias is embedded in digital health technology because of skewed data or knowledge available. According to a study [11] on trends in gender-related AI in medical research, the number of publications has steadily increased over time from 2001 to 2020, with a steep increase between 2016 and 2020, accounting for 77.5% (2,410 of 3,110) of all included papers (Table 1). That study also showed that the percentages of gender-related articles in medical AI research doubled from 3% to 6.5% from 2001 to 2020 in a bibliometric analysis conducted by searching Web of Science.

Despite the rapid increase in research related to medical AI, few studies have actually integrated SGBA, potentially leading to the development of more gender-biased medical AIs. Therefore, more attention should be given to research integrating sex and gender

## Awareness of sex and gender-based analysis in biomedical research in Korea and policy development

SGBA in research was formally introduced through a case study supported by the Korea Foundation for Women in Science, Engineering and Technology [12] and the Gender Summit 6 Asia-Pacific, which was held in Seoul in 2015 [13]. Subsequently, starting in 2016, the National Research Foundation supported a 5-year project reflecting sex and gender characteristics, through which various cases were accumulated and policy agendas were developed [14]. However, the awareness of SGBA in research in Korea is still low, as shown in Table 2. It is challenging that only 1% of principal investigators who participated in the investigation and analysis of national research and development (R&D) projects considered SGBA to be applicable to their projects [6].

Although funding policy is a major component of infrastructure for research and innovation to encourage the implementation of SGBA into the entire research process, institutions also play a pivotal role in developing research methodology to integrate SGBA and providing expertise to future generations [4]. As funding policy in Korea is based on the Master Plan of Science and Technology (S&T), the integration of SGBA has to be introduced into the Master Plan of S&T, which is established by the Ministry of Science and ICT. A consideration of mid- and long-term policy objectives and directions for S&T development every 5 years is required by the Framework Act on Science and Technology. The legal basis for the integration of SGBA into the full process of research and innovation is well established via the amendment of the Framework Act on Science and Technology, enacted on April 20, 2021 [15]. The amendment of the Framework Act introducing sex and gender consideration was made possible by the proposal submitted by the Honorary Congressman Seounglae Jo after a consensus was fully developed during the Gender Summit Global for SDGs hosted by the Korea Center of Gendered Innovations for Science and Technology Research (GISTeR), which was held in Seoul in 2020 [16]. This event emphasized the role of SGBA in the entire process of research and development as one of the main tools for inclusive innovations for sustainable development. Before the summit, a similar bill was submitted in 2018 but did not pass until the end of the 20th National Assembly in June 2020. Since 2016, GISTeR has held various activities including expert forums and roundtable discussions with the National Assembly to raise publicity.

The content of the amendment of the Framework Act on Science and Technology introducing SGBA is shown in Table 3, and its impacts are described below.

According to Article 7, 15-4, the Master Plan shall include the implementation of S&T to enhance social values in consideration of characteristics such as sex and gender and their intersectional factors. Based on Article 7, 15-4, in the Fifth Master Plan of S&T the paragraph “securing a policy base for the integration of SGBA into research” is reflected in Task 2 <Improvement of Research Environment to Increase Autonomy and Creativity> in Strategy 1 <Advancement of Science and Technology System for Qualitative Growth>. Based on these legal and institutional foundations, SGBA shall be reflected in the annual implementation plan starting in 2023 and there should be many research projects on integrating SGBA. However, despite this legal framework, in reality these efforts may be very limited because the implementation of SGBA is left to the researchers’ autonomy. This is in contrast to what experts in gendered innovations recommend as the most effective approach for SGBA (i.e., the mandatory implementation of SGBA by funding agencies).

Article 14 (3), ensuring the analysis of characteristics such as sex/gender in the Technology Assessment and Evaluation, could be regarded as the most advanced and innovative policy at a worldwide level. This policy stipulates that SGBA shall be applied in the whole process of technology innovation and create new value through new products and services for both men and women by considering sex and gender differences wherever applicable. Furthermore, the target of technology impact assessment is emerging technologies in the future, and sex and gender could be often overlooked or ignored because of a lack of understanding of the impact of sex and gender in humans. For example, the target of the 2022 technical impact assessment in Korea is synthetic biology [17], which is generally considered as having nothing to do with sex and gender by synthetic biologists, because their biomaterial has no sex. However, when synthetic biomaterial is used, it can affect the environment and elicit subsequent effects in both men and women. There is much scientific evidence [18,19] for sex differences in chemical toxicity and gender differences in the acceptability of new technology. Thus, the methodology of technology impact assessment for any targets integrating SGBA should be developed at the global level.

Finally, Article 26-2 deals with an inclusive approach to data management, analysis, and utilization to measure the development of SGBA implementation in research and innovation. Data management reducing gender bias would be a major challenge in developing health technology, as well as in research on the integration of medical technology with information and communication technology.

As described, the development of SGBA in biomedicine, health, medical AI, and emerging technologies may not be suitable to leave to the autonomy of researchers because the available scientific evidence indicates that research should integrate SGBA. Thus, the Act on the Performance Evaluation and Management of National Research and Development Project further fortified SGBA on June 29, 2021 [20] to promote SGBA research (Table 4).

Article 3 ⑦ ensures that evaluation and monitoring of the development of SGBA in research will take place at the national level, even though the actual implementation of SGBA is left to researchers’ autonomy or discretion.

## Concluding remarks

As reviewed above, the EU has been reflecting the gender dimension in research policies since 2013, and the US NIH puts research excellence at the forefront and has mandated the submission of research proposals with sex as a variable in research on vertebrate animals and humans since 2016. Moreover, the Government of Canada’s Health Portfolio uses ‘Sex- and Gender-Based Analysis Plus (SGBA Plus)’ to develop, implement, and evaluate not only the Health Portfolio’s research, but also surveillance, legislation, policies, regulations, programs, services, and other initiatives related to national health policies. The objective of this policy is to strengthen the integration and application of SGBA Plus in all Health Portfolio’s activities to advance equity, diversity, and inclusion [21]. Korea has also specified that SGBA should be reflected in R&D through an amendment of the Framework Act on Science and Technology from 2021 onwards.

Despite the introduction and dissemination of these relevant policies and acts to promote SGBA research, the integration of SGBA in actual research settings has been slow. An important message for individual researchers, funding agencies, and scholarly journals aiming to expand SGBA are to establish a research culture in which all researchers and science policy experts trust that gendered innovations are for “Better Science and Better Life” for both women and men, as well as for research excellence. Inclusive innovation could be accelerated by developing a sound methodology to practice SGBA by improving awareness that SGBA is meant to create better knowledge throughout the whole research process.

I hope that readers will be mindful of the recent global movements and the legislative trends within Korea aimed at promoting SGBA, and thus be encouraged to take part in gendered innovations.

## Figures and Tables

**Table 1.** Trends in gender-related research in medical artificial intelligence

Year	Number of papers	Proportion (%)
2001–2005	54	1.7
2006–2010	153	4.9
2011–2015	493	15.9
2016–2020	2,410	77.5
Total	3,110	100

**Table 2.** Principal investigators’ responses on the applicability of SGBA among 2019 national R&D projects

Applicability of SGBA	Number of projects		Funding size	
	Number	Proportion (%)	Funding (million USD)	Proportion (%)
Not applicable	3,253	91.9	1,061	94.3
Applicable	37	1.0	6	0.6
No answer	250	7.1	57	5.1
Total	3,540	100	1,124	100

R&D, Research and development; SGBA: sex and gender-based analysis. 1 US dollar (USD)=1,299 Korean won.

**Table 3.** Integration of sex and gender into the Framework Act on Science and Technology

Articles	Content
Article 7 (Master Plans for Science and Technology)	15-4 Implementation of science and technology to enhance social values in consideration of characteristics such as sex and gender
Article 14 (Technology Assessment and Evaluation)	(3) When conducting a technology impact assessment, the government should ensure that the analysis of characteristics such as sex/gender is reflected by taking into account the characteristics of the target technology.
Article 26-2 (Surveys and Analysis of Scientific and Technological Statistics and Indexes)	(3) When investigating and analyzing science and technology statistics and indicators, the government should reflect the characteristics of the analysis such as sex/gender by considering the characteristics of individual science and technology statistics and indicators.

**Table 4.** Integration of sex and gender into Act on the Performance Evaluation and Management of National Research and Development Projects, etc.

Article	Content
Article 3 (Basic Principles of Performance Evaluation and Performance Management)	⑦When conducting the performance evaluation, the government shall consider whether characteristics such as sex and gender were reflected, taking into account the nature of the research and development project.
